# Prediction of acute kidney injury in mechanically ventilated patients with COVID-19-related septic shock: An exploratory analysis of non-renal organ dysfunction markers

**DOI:** 10.62838/jccm-2026-0005

**Published:** 2026-04-30

**Authors:** Jose J. Zaragoza, Jonathan S. Chávez-Iñiguez, Armando Vazquez-Rangel

**Affiliations:** Critical Care Department, Hospital H+ Queretaro,Mexico; Nephrology Department, Hospital Civil de Guadalajara Fray Antonio Alcalde, Guadalajara, Mexico; Centro Uniersitario de Ciencias de la Salud, Universidad de Guadalajara, Guadalajara, Mexico; Nephrology Department, Instituto Nacional de Cardiologia, Mexico City, Mexico

**Keywords:** acute kidney injury, organ dysfunction, acute kidney injury prediction, critical care, intensive care unit, SOFA score, APACHE II score

## Abstract

**Background:**

Acute kidney injury (AKI) is a common and serious complication in critically ill patients with non-kidney organ dysfunction. Early prediction of AKI is crucial for timely intervention and improved outcomes. This study aimed to identify readily available non-renal predictors of AKI and to develop an exploratory prediction model in a specific cohort of critically ill patients with COVID-19-related septic shock requiring mechanical ventilation.

**Materials and methods:**

This was a single-center, observational, retrospective cohort study conducted in the respiratory ICU of Hospital H+ Querétaro between April and December 2020. The study included 42 mechanically ventilated patients with septic shock secondary to SARS-CoV-2 infection and non-kidney organ dysfunction. AKI was defined using the KDIGO criteria. Trend analysis, bivariate and multivariate linear regression, were used to identify predictors of AKI and severe AKI.

**Results:**

AKI occurred in 23 (54.8%) patients, with 6 (14.3%) developing severe AKI. Trend analysis revealed differences in norepinephrine dose, hemoglobin, and lactate trends between groups. A simplified logistic regression model, validated internally with bootstrapping to prevent overfitting, identified a protective trend associated with higher hemoglobin levels on admission. Quantitative analysis of a forecasting model for daily renal function showed moderate predictive accuracy.

**Conclusions:**

This study identified several readily available non-kidney organ dysfunction variables that can predict AKI and its severity in critically ill patients with COVID-19-related septic shock. These findings may help in the early identification of at-risk patients and facilitate timely interventions to potentially improve outcomes. Further validation in larger and more diverse populations is warranted.

## Introduction

Acute kidney injury (AKI) is a complex disorder characterized by a rapid decline in kidney function, resulting in the accumulation of metabolic waste products and fluid imbalance [1]. It is a common and serious complication in critically ill patients, affecting approximately 30–50% of those admitted to intensive care units (ICU) [2]. This syndrome is associated with increased morbidity, mortality, and healthcare costs [3]. The prognosis of AKI depends on its severity and the underlying cause, with the worst outcomes observed in those requiring kidney replacement therapy (KRT), where mortality approaches 60% [4]. AKI is frequently triggered by non-renal organ dysfunction, such as sepsis [5], respiratory failure [6], and heart failure [7]. These conditions can lead to decreased kidney perfusion, inflammation, and oxidative stress, which can damage the kidneys [8]. While the KDIGO consensus criteria provide a standardized definition of AKI based on changes in serum creatinine (SCr) and urine output (UO) [9], these markers are often delayed and may lack sensitivity for early detection [10]. Trend analysis is a valuable but underexplored statistical tool for predicting the evolution of patients in the ICU and implementing early diagnostic and treatment strategies. Early prediction of AKI is crucial for timely intervention and improved outcomes. Several interventions have demonstrated effectiveness in preventing or mitigating AKI, including fluid management, hemodynamic optimization, and avoidance of nephrotoxic medications [11]. Several prediction models for AKI have been developed, but most have focused on specific populations, such as patients undergoing cardiac surgery or those with sepsis [12]. Therefore, there is a need for a prediction model that can be applied to a broader population of critically ill patients with non-kidney dysfunction. These models often incorporate various risk factors, including demographics, comorbidities, laboratory values, and clinical parameters. However, the accuracy of these models varies, and a universally accepted model for AKI prediction in all critically ill patients does not yet exist [13].

The aim of this study was to identify readily available non-renal predictors of AKI and to develop an exploratory prediction model in a specific cohort of critically ill patients with COVID-19-related septic shock requiring mechanical ventilation.

## Methods

### Study Design and Participants

This was a single-center, observational, retrospective cohort study conducted at the respiratory ICU of Hospital H+ Querétaro, a 50-bed academic private hospital with a 10-bed respiratory ICU. The study protocol was designed to align with the Strengthening the Reporting of Observational Studies in Epidemiology (STROBE) guidelines [14] and the REporting of studies Conducted using Observational Routinely collected health Data (RECORD) statement [15]. Patients admitted to the respiratory ICU between April 15, 2020, and December 31, 2020, were screened for eligibility. The inclusion criteria were adults (≥18 years) admitted to the ICU requiring invasive mechanical ventilation for acute respiratory distress syndrome (ARDS) and presenting with septic shock, according to the Sepsis-3 criteria, secondary to a confirmed SARS-CoV-2 infection. Non-kidney organ dysfunction was defined as the presence of one or more of the following: sepsis, respiratory failure, heart failure, or liver failure. Patients were excluded if they had pre-existing chronic kidney disease stage 5 (CKD) or end-stage kidney disease (ESKD), defined as an estimated glomerular filtration rate (eGFR) <15 mL/min/1.73 m^2^ or the need for dialysis, a history of solid organ transplantation, pregnancy, or missing data on key variables.

### Definitions

AKI was defined according to the Kidney Disease Improving Global Outcomes (KDIGO) criteria [16]. Severe AKI was considered when KDIGO stage 2 or 3 was met. Baseline creatinine was defined as the most recent SCr value available within 3 months prior to hospital admission. If unavailable, it was estimated using the MDRD equation, assuming a baseline GFR of 75 ml/min/1.73m^2^ [17]. Organ dysfunction was measured using the SOFA Score [18]. Sepsis and septic shock were defined according to the Sepsis-3 criteria [19]. Respiratory failure was defined as the need for mechanical ventilation [20]. Heart failure was defined as the presence of dyspnea, pulmonary edema, and elevated brain natriuretic peptide (BNP) levels [21]. Liver failure was defined as the presence of jaundice, ascites, and coagulopathy [22].

### Data Collection

Data were collected from electronic medical records using a standardized data collection form. The following data points were captured for up to 7 days of ICU stay or until ICU discharge, whichever occurred first: demographics (age, sex, race, ethnicity), comorbidities (diabetes mellitus, hypertension, coronary artery disease, heart failure, chronic obstructive pulmonary disease, liver disease), laboratory values (SCr, blood urea nitrogen, blood glucose, white blood cell count, hemoglobin, platelet count, lactate, C-reactive protein), and clinical parameters (vital signs including heart rate, respiratory rate, blood pressure, temperature, mechanical ventilation, vasopressor use, SOFA score, Acute Physiology and Chronic Health Evaluation II (APACHE II) score).

### Study Objectives

The primary objective was to validate a prediction model for AKI in a general population of critically ill adults with non-kidney organ dysfunction. The secondary objectives were to assess the incidence of AKI in this population and the mortality risk.

### Statistical Analysis

Descriptive analysis was performed to assess the normality of continuous variables using histograms, kernel density plots, the Shapiro-Wilk test, and the Skewness and Kurtosis test. Due to the predominantly non-normal distribution, continuous variables are presented as medians and interquartile ranges (IQR), while categorical variables are presented as frequencies and percentages. Comparisons between groups (severe AKI vs. no severe AKI) were performed using the Mann-Whitney U test for continuous variables and the χ^2^ test or Fisher’s exact test for categorical variables, as appropriate. Kaplan-Meier curves were generated to illustrate the time to AKI and severe AKI. Trend analysis was conducted by visual inspection of box plots to identify variables with differing trends between groups. To explore early predictors of AKI while addressing concerns about model overfitting due to the sample size, the primary analysis was shifted from a complex multivariate model to a simplified logistic regression model using only two clinically relevant day-1 predictors. The stability of this model’s estimates was assessed using bootstrap validation with 1000 repetitions to generate robust 95% confidence intervals. The predictive accuracy of the initial forecasting model for daily serum creatinine and urine output was evaluated quantitatively by calculating the Mean Absolute Error (MAE) and Root Mean Squared Error (RMSE) for days 4 through 8.

### Ethical Considerations

The study was approved by the Research Ethics Committee of the Hospital Español with the number F-70-2017 and was conducted according to the Declaration of Helsinki. In adherence to this, consent was not required for this study due to its retrospective nature and the use of de-identified data.

## Results

**Fig. 1.**
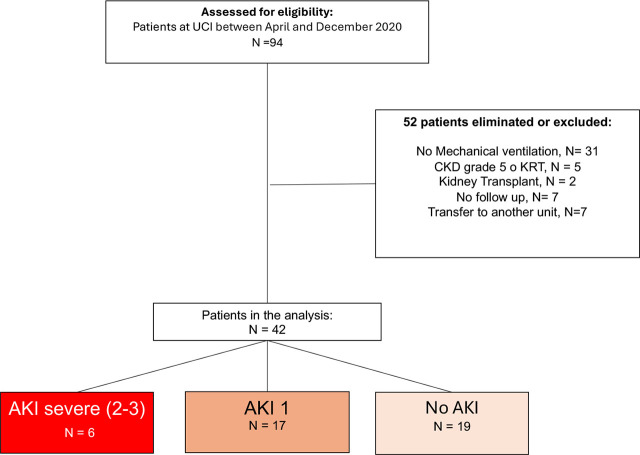
Study flowchart

**Fig. 2.**
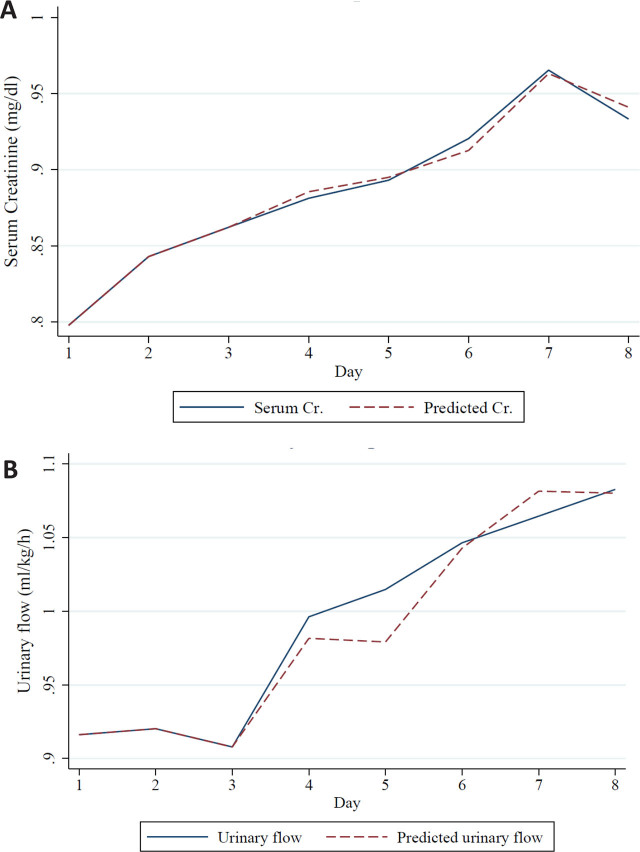
A. Creatinine prediction; B. Urinary flow prediction

Between April 15 and December 31, 2020, a total of 94 patients were screened in the respiratory ICU. Of these, 15 patients were excluded due to incomplete follow-up (n=7), initiation of KRT within the first 24 hours of ICU admission (n=1), or transfer to another hospital (n=7). Consequently, 42 patients met the inclusion criteria and were included in the final analysis. AKI, defined by the KDIGO criteria, occurred in 23 patients (54.8%). Among those with AKI, 6 patients (14.3% of the total cohort) developed severe AKI (KDIGO stage 2 or 3), as illustrated in the flowchart in Figure 1. Baseline characteristics stratified by AKI severity (no severe AKI vs. severe AKI) are presented in Table 1. The median age of the entire cohort was 53.5 years (45–61), and 36(85%) were male. The prevalence of comorbidities in the total cohort was as follows, diabetes mellitus in 13 (31%) patients, hypertension in 11 (26.1%), and obesity in 23 (54.7%). Baseline eGFR was 112 ml/min/1.73m^2^. The management of sedoanalgesia was similar between the groups. Vasopressors were required in 38 (90%) patients, and the median cumulative fluid balance on day 1 was minimal at 69.5 ml (594–478). When comparing to the non-severe AKI, the severe AKI group presented with higher median PaO_2_/FiO_2_ values (218 vs 147, p=0.01), which was also reflected in the respiratory component of the SOFA score. No patients in the included cohort required KRT during their ICU stay. The median length of hospital stay was 16.5 days, and the overall in-hospital mortality rate was 11.9% (5 out of 42 patients). The remaining baseline characteristics and the statistical comparisons between the groups are detailed in Table 1. The median time to development of any-stage AKI was 6 days (IQR 3–8). The Kaplan-Meier analysis illustrating the proportion of patients remaining free of AKI and severe AKI over the first 8 days is presented in Figure 2.

**Table 1.** Baseline characteristics of the study population

**Variable**	**No Severe AKI (36)**	**Severe AKI (6)**	**Total (42)**	**p-value**
**Baseline Characteristics**				
Age (years)	53.5 (45-60.5)	54 (49-72)	53.5 (45-61)	0.69
Male sex (%)	30 (83)	6 (100)	36 (85)	0.56
Height (cm)	171.5 (165-176.5)	172.5 (163-187)	171.5 (165-177)	0.44
Weight (kg)	84 (75.5-93)	90.5 (75-102)	85 (75-95)	0.55
Diabetes Mellitus	9 (25)	4 (66)	13 (31)	0.06
COPD	1 (3)	0	1 (2.38)	0.67
Hypertension	9 (25)	2 (33)	11 (26.1)	0.64
Obesity	19 (53)	4 (67)	23 (54.7)	0.67
OSAS	3 (8)	0	3 (7.14)	0.46
Smoking	1 (3)	0	1 (2.38)	0.67
Immunosuppression	3 (8)	0	3 (7.14)	0.46
Hypothyroidism	1 (3)	0	1 (2.38)	0.67
Hematologic syndrome	3 (8)	0	3 (7.14)	0.46
Days of evolution	9 (7-12)	8 (7-11)	9 (7-12)	0.88
Days to intubation	10 (8-13.5)	10.5 (8-14)	10 (8-14)	0.81
Baseline creatinine(mg/dl)	0.6 (0.5-0.7)	0.75 (0.6-1.2)	0.6 (0.5-0.7)	0.09
eGFR, ml/min/1.73m^2^	113 (104-125)	97 (69-118)	112 (103-124)	0.20

**Day one variables**				
Total SOFA	6 (5.5-7)	5.5 (5-6)	6 (5-7)	0.27
Respiratory SOFA	3 (3-3)	2.5 (2-3)	3 (3-3)	0.04*
Cardiovascular SOFA	3 (3-3)	3 (3-3)	3 (3-3)	0.72
Renal SOFA	0 (0-0)	0 (0-0)	0 (0-0)	0.33
Hematologic SOFA	0 (0-0)	0 (0-0)	0 (0-0)	0.68
Hepatic SOFA	0 (0-0)	0 (0-0)	0 (0-0)	0.54
Neurological SOFA	0 (0-0)	0 (0-0)	0 (0-0)	0.38
Maximum lactate	1.7 (1.4-2.4)	1.7 (1.4-2.3)	1.7 (1.4-2.3)	0.97
Minimum lactate	1.6 (1.3-1.9)	1.55 (1.2-1.8)	1.6 (1.3-1.8)	0.92
Maximum heart rate	84.5 (78.5-93.5)	86 (83-105)	84.5 (80-94)	0.56
Minimum heart rate	58 (51-63)	64.5 (52-74)	58 (52-65)	0.23
Maximum PAM	94.5 (90-100)	96 (90-98)	95 (90-99)	0.82
Minimum PAM	71 (68.5-74)	69 (63-73)	71 (68-74)	0.27
Maximum PEEP	12 (10-12)	11 (10-12)	12 (10-12)	0.52
Minimum PEEP	10 (9-11.5)	11 (10-12)	10 (9-12)	0.35
PaO_2_ / FiO_2_	147 (114-176.5)	218 (189-242)	151 (124-192)	0.01*
Sevoflurane	16 (44)	4 (66)	20 (47.62)	0.40
Fentanyl	31 (86)	6 (100)	37 (88.1)	0.33
Remifentanil	4 (11)	0	4 (9.52)	0.39
Cisatracurio/Rocuronio	14 (38)	0	14 (33.3)	0.06
Norepinephrine	32 (89)	6 (100)	38 (90.4)	0.39
Norepinephrine dose (µ/kg/h)	0.032 (0.014-0.076)	0.045 (0.019 – 0.085)	0.032 (0.018 – 0.081)	0.67
Fluid balance (ml)	10.5 (-619-414)	356 (153-1663)	69.5 (-594-478)	0.22
Urinary output (ml)	1687.5 (995-2200)	1421 (750-2130)	1652.5 (980-2130)	0.56
Hb	15.3 (14.1-16.4)	14.2 (13.9-16.5)	15.1 (13.9-16.5)	0.57
Platelets	233 (200-312)	269 (199-321)	233.5 (199-317)	0.91
Leukocytes	11.36 (6.94-13.73)	11.62 (7.49-13.39)	11.36 (7.15-13.7)	0.71
Neutrophil	9.57 (5.9-11.77)	10.52 (5.83-11.78)	9.71 (5.83-11.78)	0.73
Lymphocytes	0.9 (0.7-1.26)	0.86 (0.57-1)	0.91 (0.66-1.23)	0.43
Creatinine	0.75 (0.7-0.85)	0.9 (0.6-1.2)	0.75 (0.7-0.9)	0.52
BUN	16 (13-21.5)	17 (12-24)	16 (13-22)	0.85
Total bilirubin	0.65 (0.44-0.83)	0.79 (0.38-0.95)	0.65 (0.44-0.83)	0.68
AST	47.5 (35-66)	42 (32-51)	46 (34-66)	0.59
ALT	42.5 (27-54)	54.5 (35.5-69)	46 (27-56)	0.41
LDH	456 (390-519)	376 (323-452)	444 (369-513)	0.13
Albumin	3 (2.7-3.4)	3.3 (3-3.6)	3 (2.8-3.5)	0.24
Na	139.5 (137-142)	140 (139-141)	140 (137-142)	0.85
K	4.1 (3.8-4.6)	4.4 (4.1-4.8)	4.15 (3.8-4.6)	0.24
Ca	8.5 (8.1-8.7)	8.8 (8.7-8.9)	8.5 (8.1-8.8)	0.23
Procalcitonin	0.16 (0.7-0.44)	0.33 (0.09-0.56)	0.16 (0.07-0.55)	0.73
D dimer	1095.9 (566-1740)	995 (665-3592)	1095.9 (607-1764)	0.61
Ferritin	1282 (649-2273)	742 (625.3-792)	1070.5 (625.3-2273)	0.42
C-reactive protein	23 (17.8-36.6)	17.75 (11.25-30.95)	23.4 (17-36.6)	0.32
Fibrinogen	706 (641-782)	635 (528-807)	706 (583-782)	0.86

**Outcomes**				
Delirium	13 (36)	1 (17)	14 (33.3)	0.64
VAP	18 (50)	2 (33)	20 (47.62)	0.66
Antipsychotics	19 (52)	3 (50)	22 (52.38)	0.90
MV time (hours)	194 (137-276)	152 (114-218)	190 (125-241)	0.86
Reintubation	6 (17)	1 (17)	7 (16.6)	1.0
Propofol days	7 (4-8)	6.5 (5-8)	7 (4-8)	0.91
Opioid days	7 (5.5-8)	6.5 (5-8)	7 (5-8)	0.74
Days of dexmedetomidine	8 (6.5-8)	7 (6-8)	8 (6-8)	0.58
Neuromuscular blocker days	4.5 (3-7.5)	2 (2-3)	4 (2-7)	0.03*
Days in ICU or hospital	17 (14-24.5)	15 (11-19)	16.5 (13-23)	0.45
Death in hospital	4 (11)	1 (16)	5 (11.9)	0.69

AKI, acute kidney injury; AST, aspartate aminotransferase; ALT, alanine aminotransferase; BUN, blood urea nitrogen; eGFR, estimated glomerular filtration rate; Ca, calcium; COPD, chronic obstructive pulmonary disease; Hb, hemoglobin; ICU, intensive care unit; K, potassium; LDH, lactate dehydrogenase; MV, mechanical ventilation; Na, sodium; PaO_2_/FiO_2_, Partial pressure of oxygen in arterial blood, / Fraction of inspired oxygen; PEEP, positive end-expiratory pressure.

### Trend Analysis and Linear Regression

Visual inspection of box plots revealed differences in the trends of norepinephrine dose, hemoglobin levels, and maximum lactate levels between the groups with and without AKI (Supplementary material A). Analysis of deltas (changes in variables over the first 3 days of ICU stay) showed significant correlations between changes in norepinephrine dose and hemoglobin levels and subsequent changes in both SCr and urine output, as summarized in Table A and illustrated in Figure B of Supplementary material. Specifically, changes in norepinephrine dose from day 2 to 3 and from day 1 to 3 showed significant positive correlations with changes in SCr from day 3 to 4. Similarly, changes in the hemoglobin from day 2 to 3 and from day 1 to 3 showed significant negative correlations with changes in SCr from day 3 to 4 and day 1 to 3, respectively. Additionally, changes in hemoglobin from day 2 to 4 showed a significant positive correlation with changes in urine flow from day 2 to 4.

### Forecasting Accuracy and Simplified Predictive Model

**Fig. 3.**
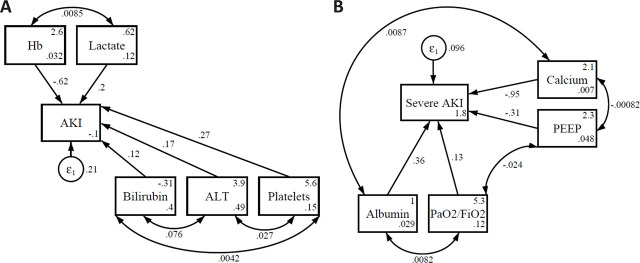
Path analysis illustrating direct and indirect clinical and biochemical contributors to AKI (A) and severe AKI (B).

**Fig. 4.**
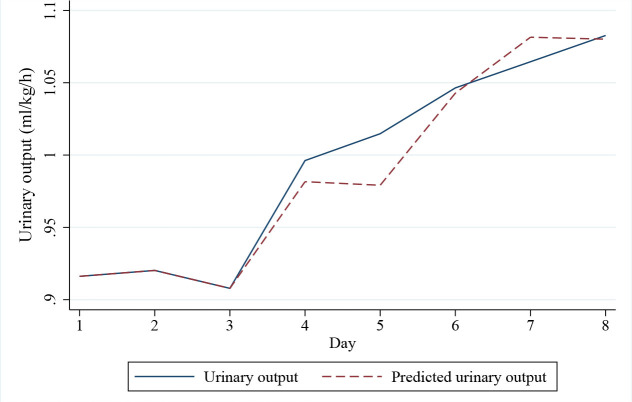
Urinary output prediction

Forecasting plots illustrating the predicted and observed trajectories of serum creatinine and urinary output are presented in Figure 3 and 4, respectively. The model’s predictive accuracy was assessed quantitatively from day 4 to day 8. For serum creatinine, the average Mean Absolute Error (MAE) from day 4 to 8 was 0.177 mg/dL, and the Root Mean Squared Error (RMSE) was 0.211 mg/dL. For urinary output, the MAE was 0.403 mL/kg/h, and the RMSE was 0.505 mL/kg/h, indicating moderate predictive performance.

To develop a more robust and statistically valid predictive model for the development of any-stage AKI, a simplified logistic regression was performed. This model, detailed in Table 2, included first-day norepinephrine dose and hemoglobin levels as predictors. Hemoglobin showed a protective trend, with each 1 g/dL increase associated with a 30% reduction in the odds of developing AKI, though this did not reach statistical significance (OR 0.70; 95% CI 0.45–1.03; p=0.085). The confidence intervals were derived from a 1000-repetition bootstrap analysis to ensure model stability.

**Table 2.** Simplified logistic regression model with bootstrapped confidence intervals for the prediction of AKI.

**Predictor (Day 1)**	**Odds Ratio (OR)**	**Bootstrap 95% CI**	**P-value**
Norepinephrine Dose (µg/kg/min)	1.02	0.95 – 1.10	0.486
Hemoglobin (g/dL)	0.70	0.45 – 1.03	0.085

## Discussion

### Main findings

In this cohort of critically ill patients admitted to the respiratory ICU with a diagnosis of septic shock secondary to SARS-CoV-2 infection and requiring mechanical ventilation, we evaluated the role of changes in non-kidney organ dysfunction in predicting the development of AKI. Our findings identified a significant association between the respiratory component of the SOFA score on the first day of admission and the subsequent development of AKI, as well as with the number of days requiring neuromuscular blocking agents.

We developed prediction models for AKI and observed that hemoglobin levels and norepinephrine dose were variables with the strongest predictive power. Specifically, for SCr, maximum PEEP, total bilirubin, and LDH were also retained in the multivariate model, while for urine output, ALT levels were additionally significant. Furthermore, using structural equation modeling, we constructed a clinically plausible path diagram for AKI prediction, which included hemoglobin, lactate, total bilirubin, ALT, and platelet count for overall AKI, and calcium, PEEP, albumin, and PaO_2_/FiO_2_ ratio for severe AKI.

Previous studies by Flechet et al. [23] and Erdfelder et al. [24] have explored the trends of SCr to predict AKI. However, the inherent limitations of SCr as a late and often erratic marker of AKI contrast with our approach, which focused on readily available, non-kidney organ dysfunction variables in the ICU setting. The clinical relevance of our findings lies in the potential to predict AKI and its severity by observing the trends of these variables, allowing for the implementation of timely interventions aimed at mitigating this risk and potentially improving patient outcomes.

### Comparison to previous studies

All patients in our study presented with sepsis, required mechanical ventilation, and the majority necessitated vasopressor support at some point during their ICU stay. Sepsis is a well-established primary factor associated with AKI in critically ill patients both within and outside the ICU. Our findings align with previous research identifying these factors as significant risk factors for AKI [16,25,26,27]. While age is also a recognized risk factor for AKI, with older patients often having more comorbidities and reduced physiological reserve [28,29], our cohort had a relatively narrow age range, which might explain why it did not emerge as a significant independent predictor in our models. Similarly, diabetes mellitus and hypertension are known to contribute to AKI through microvascular damage and decreased renal function [30], and their prevalence in our cohort was notable, suggesting they contribute to the overall risk but may not have been the primary differentiating factors in our prediction models within this specific population of septic, mechanically ventilated COVID-19 patients. Mechanical ventilation itself can impair kidney perfusion and increase intrathoracic pressure, thus contributing to AKI risk [31]. The SOFA score, as a measure of overall organ dysfunction, has consistently been associated with an increased risk of AKI [32,33], which is reflected in our findings where the respiratory component of the SOFA score showed an early association with AKI development.

The identification of hemoglobin as a potential protective factor is noteworthy. Anemia is an established risk factor for AKI [34], primarily by reducing renal oxygen delivery and rendering the kidneys more susceptible to hypoxic injury [35], a risk that is magnified in the context of shock. The trend observed in our simplified model reinforces the critical role of renal oxygenation. While our primary model was simplified, the variables identified in our initial exploratory analyses, such as LDH and platelets, may hint at underlying mechanisms. SARS-CoV-2 is known to induce significant endothelial dysfunction, a prothrombotic state, potentially leading to microthrombi in the renal vasculature leading to AKI [36].

### Strengths and limitations

A key strength of our study is that we reached the pre-calculated sample size required for both multivariate linear regression and structural equation modeling, enhancing the statistical power of our analyses. To the best of our knowledge, this is the first study to analyze the evolution and changes in non-kidney organ dysfunction variables to predict severe AKI using the concept of organ interaction as a theoretical framework in this specific population of critically ill patients with COVID-19-related septic shock. The relative homogeneity of our patient cohort in terms of baseline characteristics, driven by the inclusion criteria, suggests that we studied a group with similar clinical behaviors, potentially allowing for more accurate prediction within this context.

Our study also has several limitations. The retrospective design inherently carries the risk of potential biases in data collection and availability. We did not include all potential risk factors for AKI that have been described in the literature, which might have improved the predictive power of our models. Furthermore, our study was conducted in a single center, which may limit the generalizability of our findings to other populations and settings. The relatively small sample size, coupled with the large number of variables analyzed, could potentially impact on the reliability of our multivariate models. It is also important to note that our results are specific to mechanically ventilated COVID-19 patients treated during the initial wave of the pandemic in Mexico, which may limit their applicability to the general ICU population or even to the current landscape of SARS-CoV-2 respiratory infections. Finally, the structural equation modeling approach, while valuable for exploring complex relationships, relies on a pre-specified theoretical structure, which could introduce a degree of dependence on the researcher’s initial conceptualization. Additionally, we could not systematically account for bacterial coinfections as a potential confounder, as comprehensive microbiological data such as blood cultures were not available for all patients.

Despite these limitations, the clinical implication of our results is the potential to use this prediction model to identify patients at higher risk of developing AKI early in their ICU course, allowing for timely implementation of preventive strategies. Early interventions, such as optimized fluid management, hemodynamic support, and careful avoidance of nephrotoxic medications, may help to prevent or mitigate AKI in this vulnerable population. Our model could also serve as a valuable tool for stratifying patients in future clinical trials evaluating novel therapies for AKI in the context of severe viral infections and septic shock.

### Future Directions

Future research should focus on validating our prediction model in a larger and more diverse population of critically ill patients, including those with different etiologies of sepsis and respiratory failure. Developing a more comprehensive prediction model that incorporates a broader range of potential risk factors for AKI would also be beneficial. Additionally, prospective studies are needed to evaluate the impact of using our prediction model to guide clinical decision-making and its effect on relevant clinical outcomes, such as the incidence and severity of AKI, the need for renal replacement therapy, and overall mortality.

## Conclusion

Our prediction model, based on readily available clinical and laboratory data, can accurately predict AKI and it severity in critically ill ventilated patients with non-kidney organ dysfunction. This model can be used to identify patients at risk and allow for timely intervention, potentially improving outcomes.
